# Supplementary material for: Antimicrobial Activity and Mode of Action of N-Heterocyclic Carbene Silver(I) Complexes
Source: Molecules. 2024 Dec 28;30(1):76. doi: 10.3390/molecules30010076 (PMC11722542; doi:10.3390/molecules30010076)
Supplement: Supplementary file 1 [file molecules-30-00076-s001.zip › molecules-3268972-supplementary.pdf]

Antimicrobial activity and mode of action of N-heterocyclic carbene silver (I) complexes

Giusy Castagliuolo[1], Michela Di Napoli[1], Tshering Zangmo[2], Joanna Szpunar[2], Luisa Ronga[2], Anna Zanfardino[1]*, Mario Varcamonti[1,⁋], and Diego Tesauro [3,⁋]

Contents:

1. *2-(2-acetamidoethyl)-2H-imidazo[1,5-a]pyridin-4-ylium silver (I) chloride (5MC)*
   ESI Mass Spectrum

2. *2-(2-acetamidoethyl)-2H-imidazo[1,5-a]pyridin-4-ylium silver (I) chloride (5MC)*
   FT IR Spectrum

3. *2-(2-acetamidoethyl)-2H-imidazo[1,5-a]pyridin-4-ylium silver (I) chloride (5MC)*
   ^{1}H NMR Spectrum

4. *2-(2-acetamidoethyl)-2H-imidazo[1,5-a]pyridin-4-ylium silver (I) chloride (5MC)*
   ^{13}C NMR Spectrum

5. *2-pyridin- N-(2-ethylacetylamido)-2-yl-2-imidazole Hexafluorophosphate. (5BC)*
   ^{31}P NMR Spectrum

6. *2-pyridin- N-(2-ethylacetylamido)-2-yl-2-imidazole Hexafluorophosphate. (5BC)*
   ^{19}F NMR Spectrum

7. *2-(2-acetamidoethyl)-2H-imidazo[1,5-a]pyridin-4-ylium silver (I) chloride (Ag5MC)*
   ESI Mass Spectrum

8. *2-(2-acetamidoethyl)-2H-imidazo[1,5-a]pyridin-4-ylium silver (I) chloride (Ag5MC)*
   FT IR Spectrum

9. *2-(2-acetamidoethyl)-2H-imidazo[1,5-a]pyridin-4-ylium silver (I) chloride (Ag5MC)*
   ^{1}H NMR Spectrum

10. *2-pyridin- N-(2-ethylacetylamido)-2-yl-2-imidazole silver (I)Hexafluorophosphate (Ag5BC).*
    ^{13}C NMR Spectrum

11. *2-pyridin- N-(2-ethylacetylamido)-2-yl-2-imidazole silver (I)Hexafluorophosphate (Ag5BC).*
    ESI Mass Spectrum

12. *2-pyridin- N-(2-ethylacetylamido)-2-yl-2-imidazole silver (I)Hexafluorophosphate (Ag5BC).*
    FT IR Spectrum

13. *2-pyridin- N-(2-ethylacetylamido)-2-yl-2-imidazole silver (I)Hexafluorophosphate (Ag5BC).*
    ^{1}H NMR Spectrum

14. *2-pyridin- N-(2-ethylacetylamido)-2-yl-2-imidazole silver (I)Hexafluorophosphate (Ag5BC).*
    ^{13}C NMR Spectrum

15. *Bromo[1,3-diethyl-4,5-bis(4-methoxyphenyl)imidazol-2-ylidene]silver(I) (Ag4MC)*
ESI Mass Spectrum ($M-AgBr^+$)

16. *Bromo[1,3-diethyl-4,5-bis(4-methoxyphenyl)imidazol-2-ylidene]silver(I) (Ag4MC).*
^{1}H NMR Spectrum

17. *Bis[1,3-diethyl-4,5-bis(4-methoxyphenyl)imidazol-2-ylidene]silver(I) (Ag4BC)*
ESI Mass Spectrum ($M-MAgBr^+$)

18. *Bis[1,3-diethyl-4,5-bis(4-methoxyphenyl)imidazol-2-ylidene]silver(I) (Ag4BC).*
^{1}H NMR Spectrum

Mass spectrum of 2-(2-acetamidoethyl)-2H-imidazo[1,5-a]pyridin-4-ylium chloride (ligand 5MC)

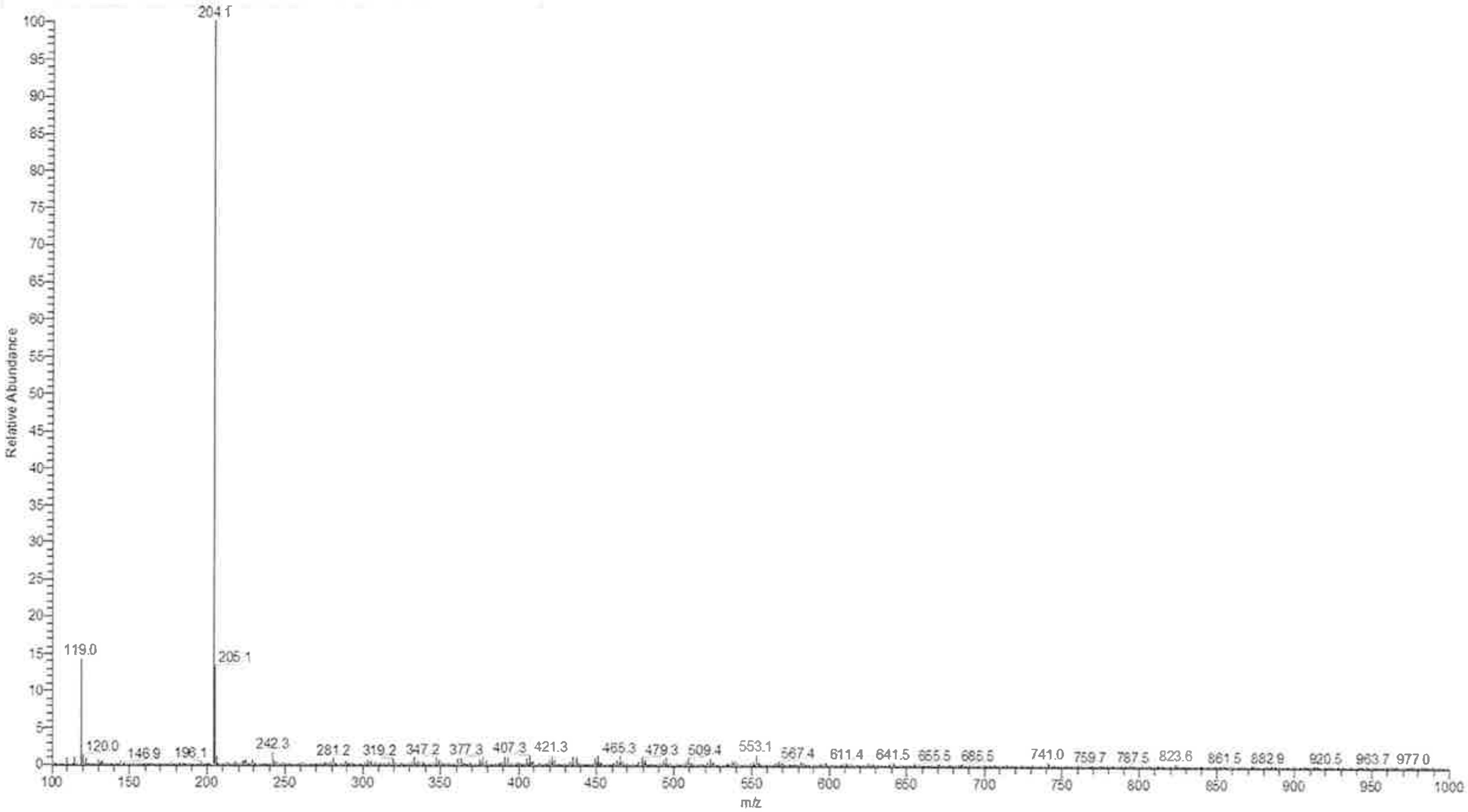

IR Spectrum of of 2-(2-acetamidoethyl)-2H-imidazo[1,5-a]pyridin-4-ylium chloride (ligand 5MC)

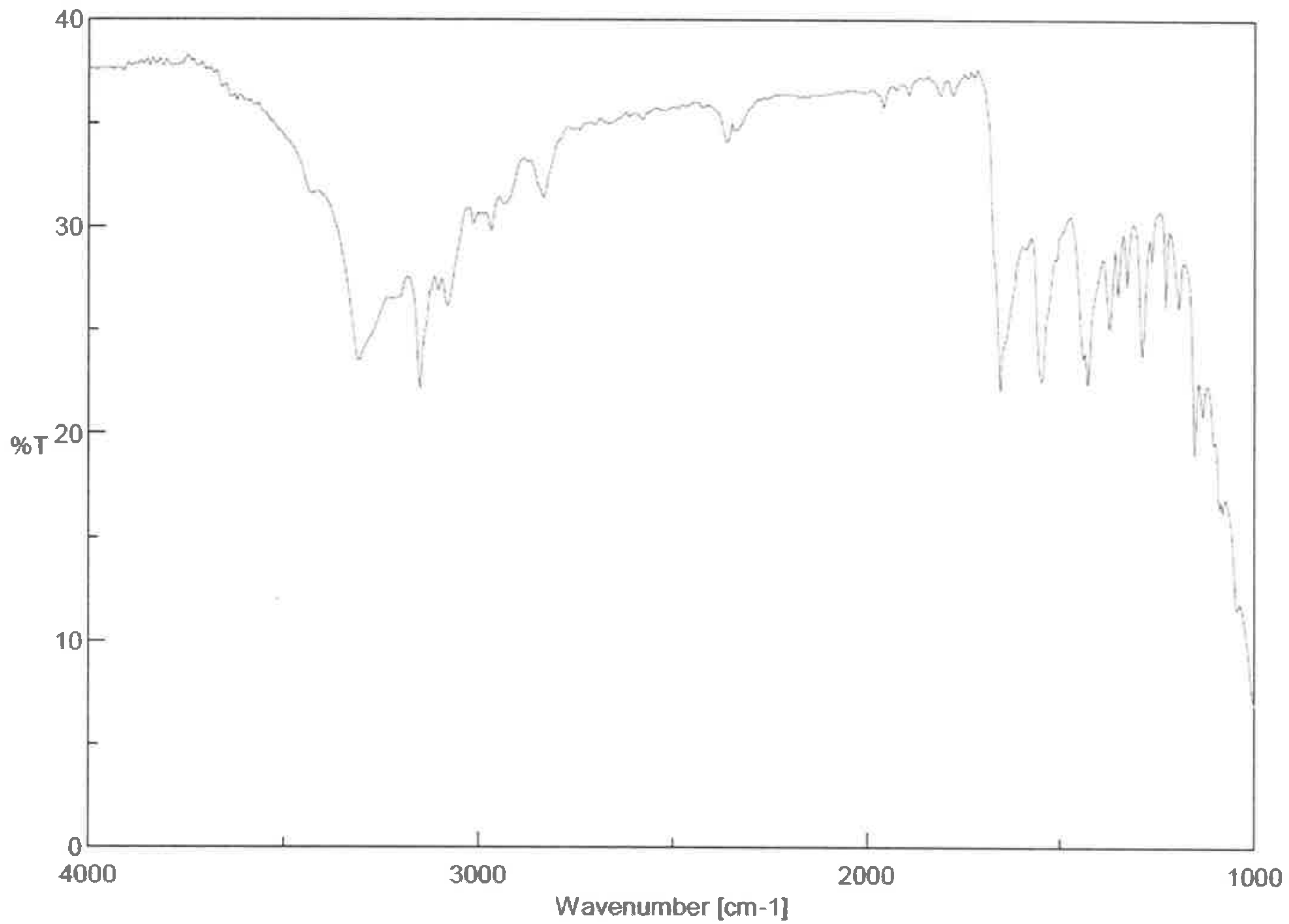

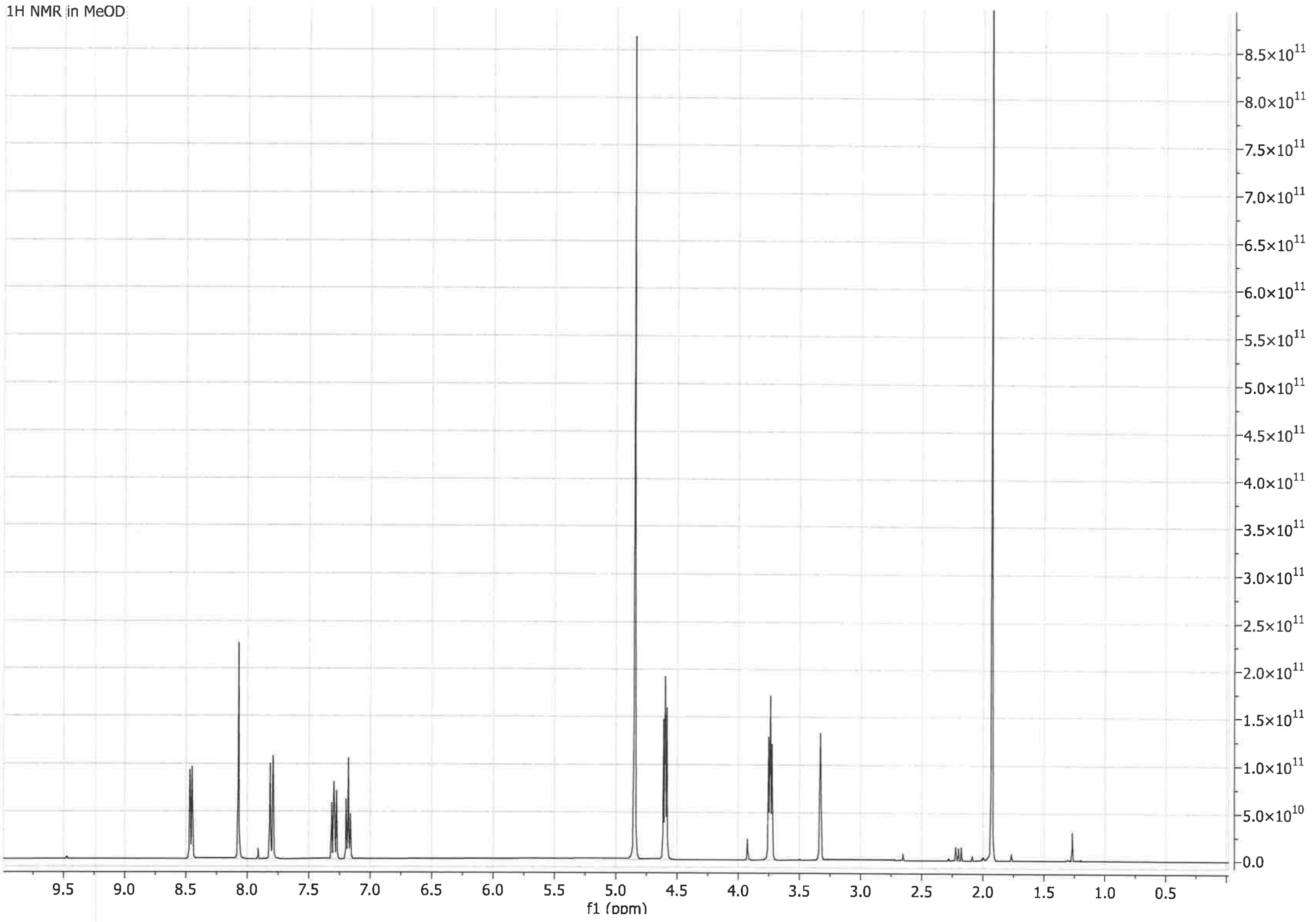
1H NMR in MeOD
9.5 9.0 8.5 8.0 7.5 7.0 6.5 6.0 5.5 5.0 4.5 4.0 3.5 3.0 2.5 2.0 1.5 1.0 0.5
f1 (ppm)
8.5×10^11
8.0×10^11
7.5×10^11
7.0×10^11
6.5×10^11
6.0×10^11
5.5×10^11
5.0×10^11
4.5×10^11
4.0×10^11
3.5×10^11
3.0×10^11
2.5×10^11
2.0×10^11
1.5×10^11
1.0×10^11
5.0×10^10
0.0

13C NMR in MeOD

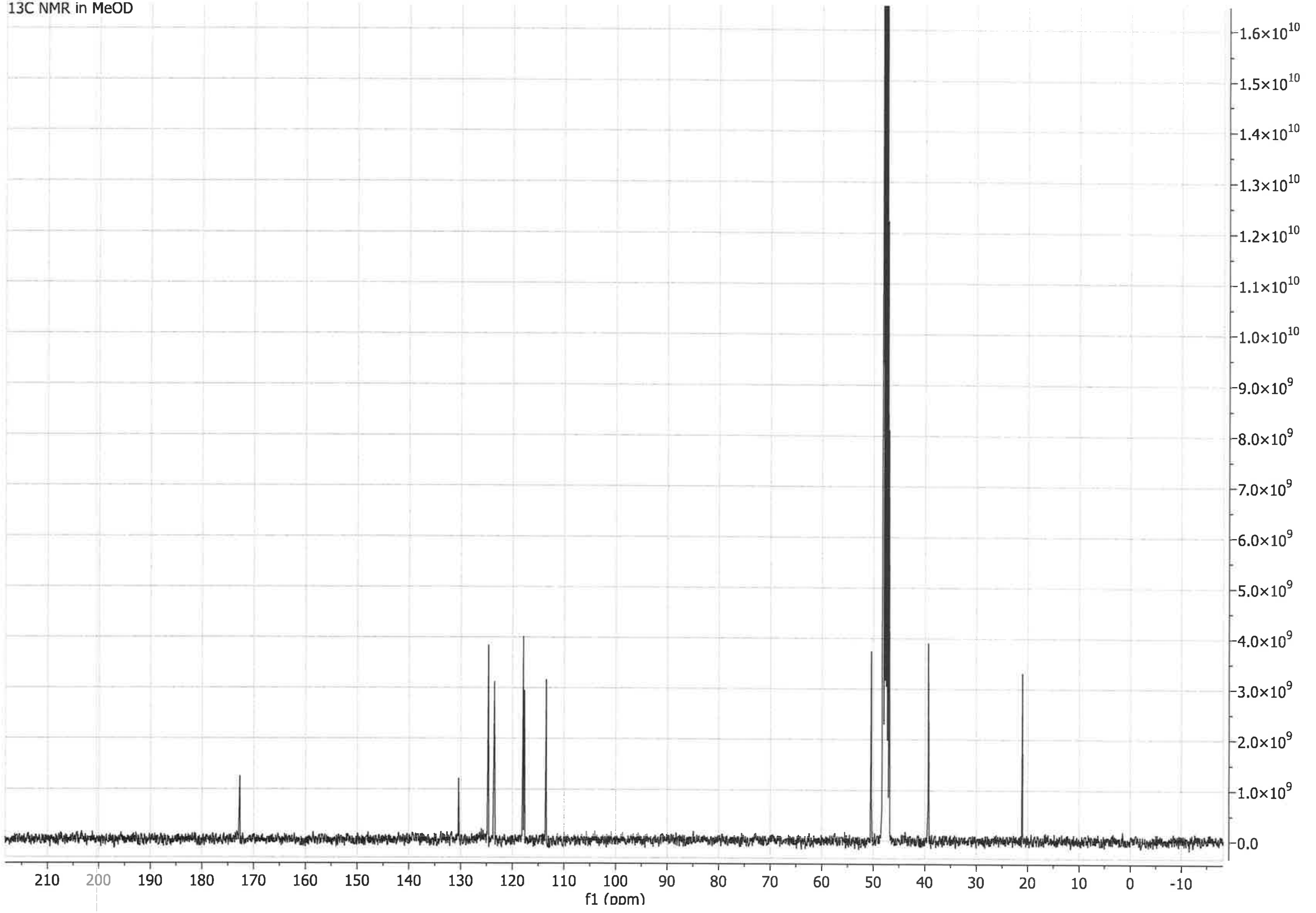

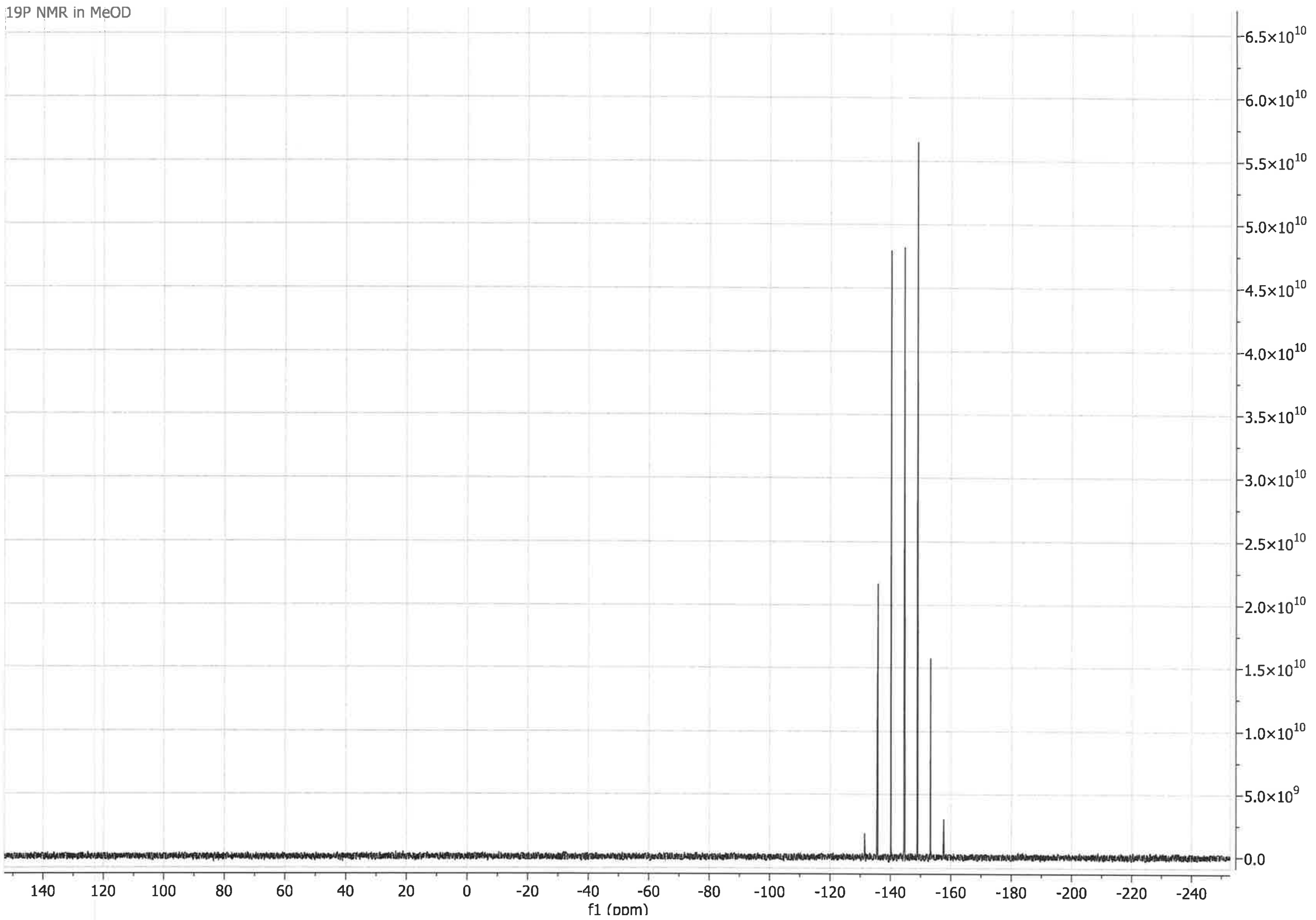
19P NMR in MeOD
6.5×10^10
6.0×10^10
5.5×10^10
5.0×10^10
4.5×10^10
4.0×10^10
3.5×10^10
3.0×10^10
2.5×10^10
2.0×10^10
1.5×10^10
1.0×10^10
5.0×10^9
0.0
140 120 100 80 60 40 20 0 -20 -40 -60 -80 -100 -120 -140 -160 -180 -200 -220 -240
f1 (ppm)

19F NMR in MeOD

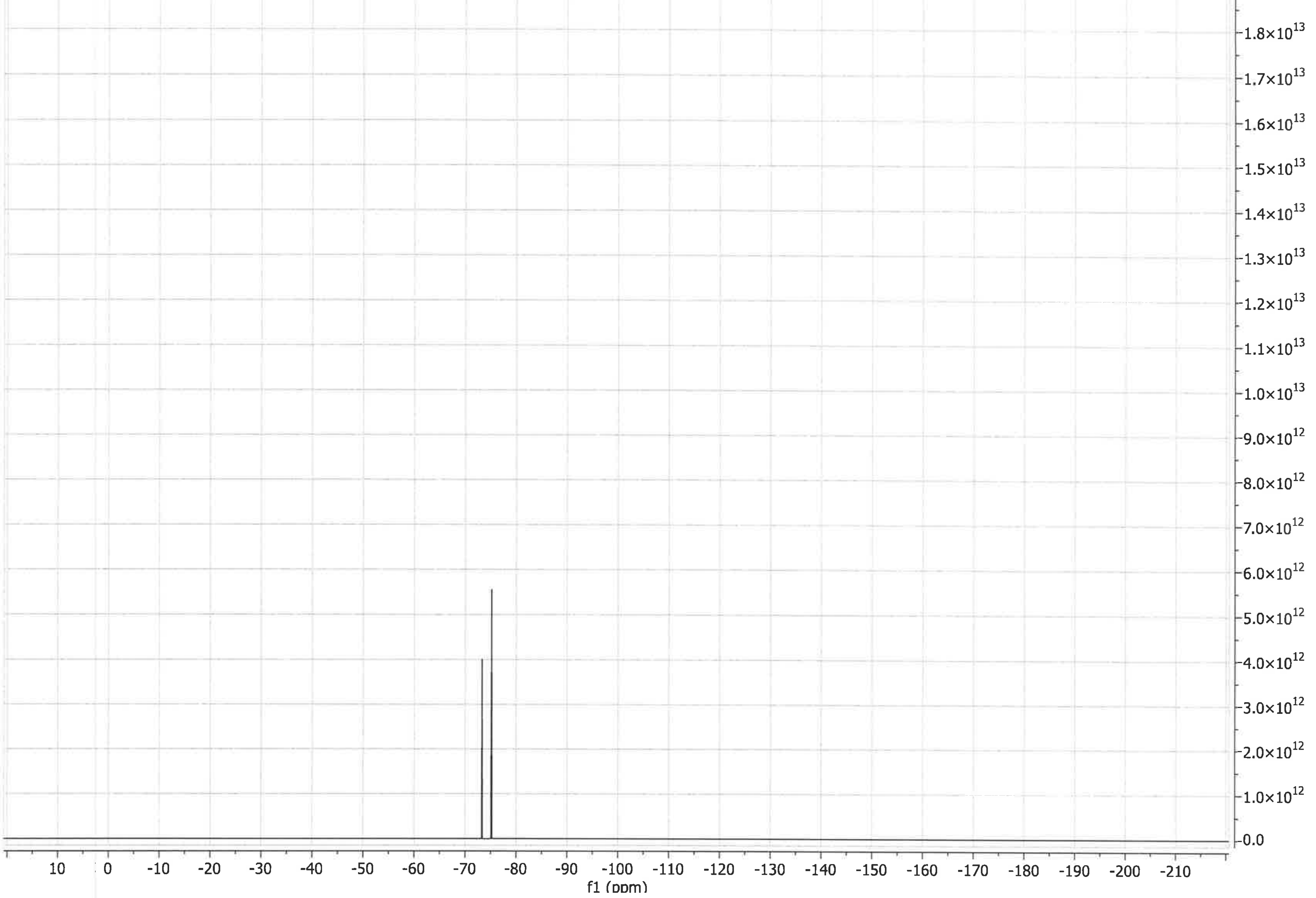

Mass spectrum of 2-(2-acetamidoethyl)-2H-imidazo[1,5-a]pyridin-4-ylium silver (I) chloride (Ag5MC)

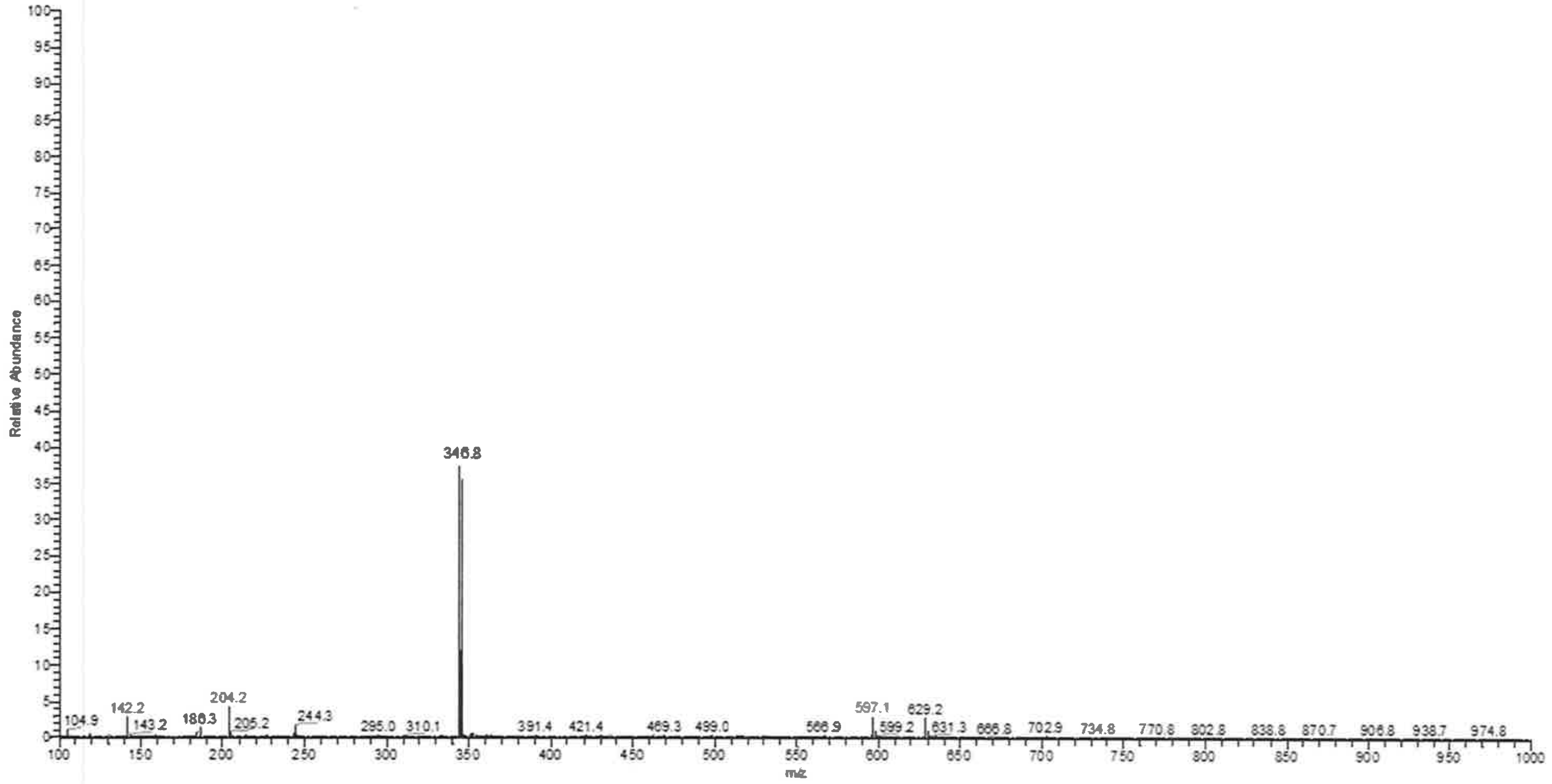

IR spectrum of 2-(2-acetamidoethyl)-2H-imidazo[1,5-a]pyridin-4-ylium silver (I) chloride (Ag5MC)

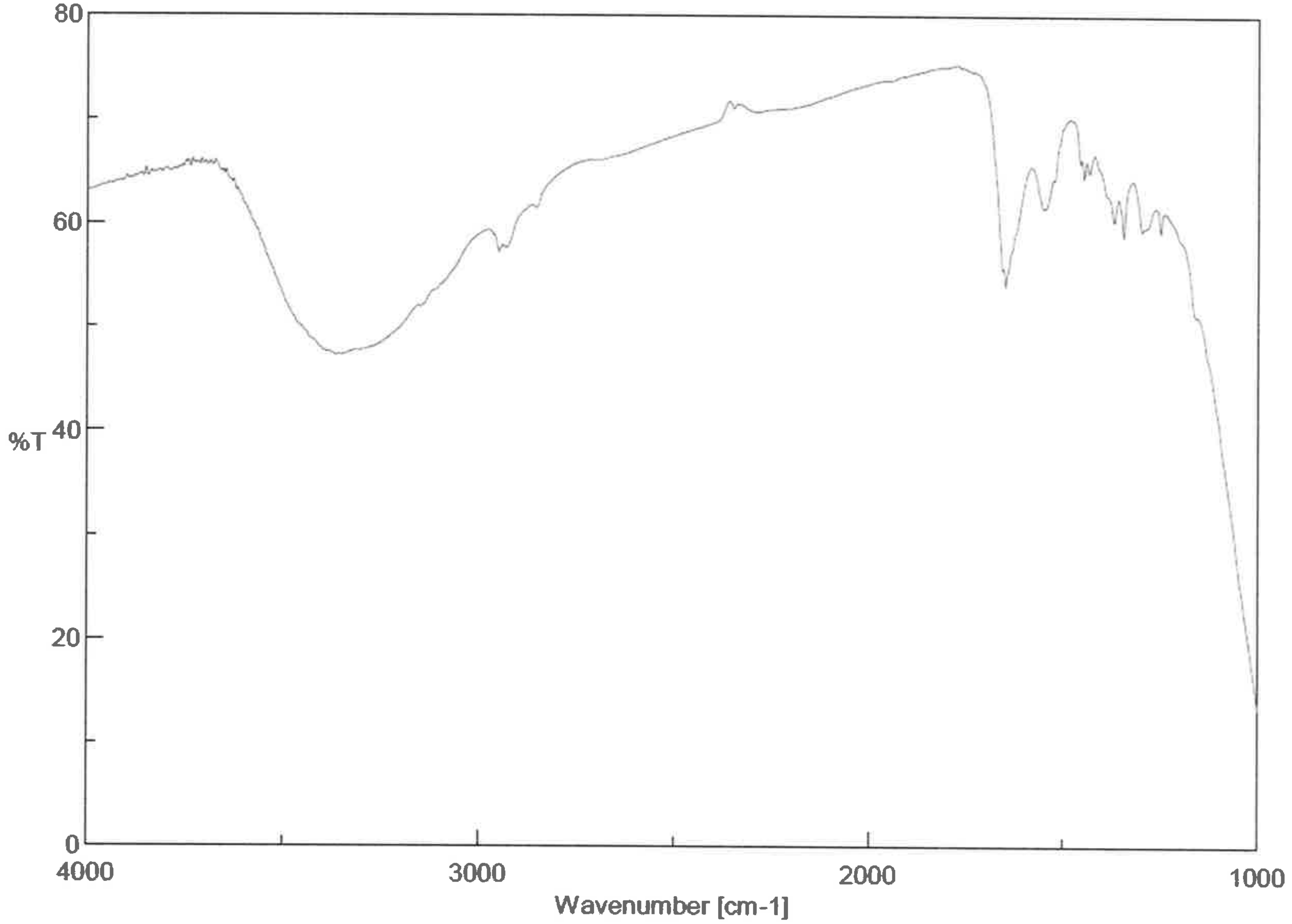

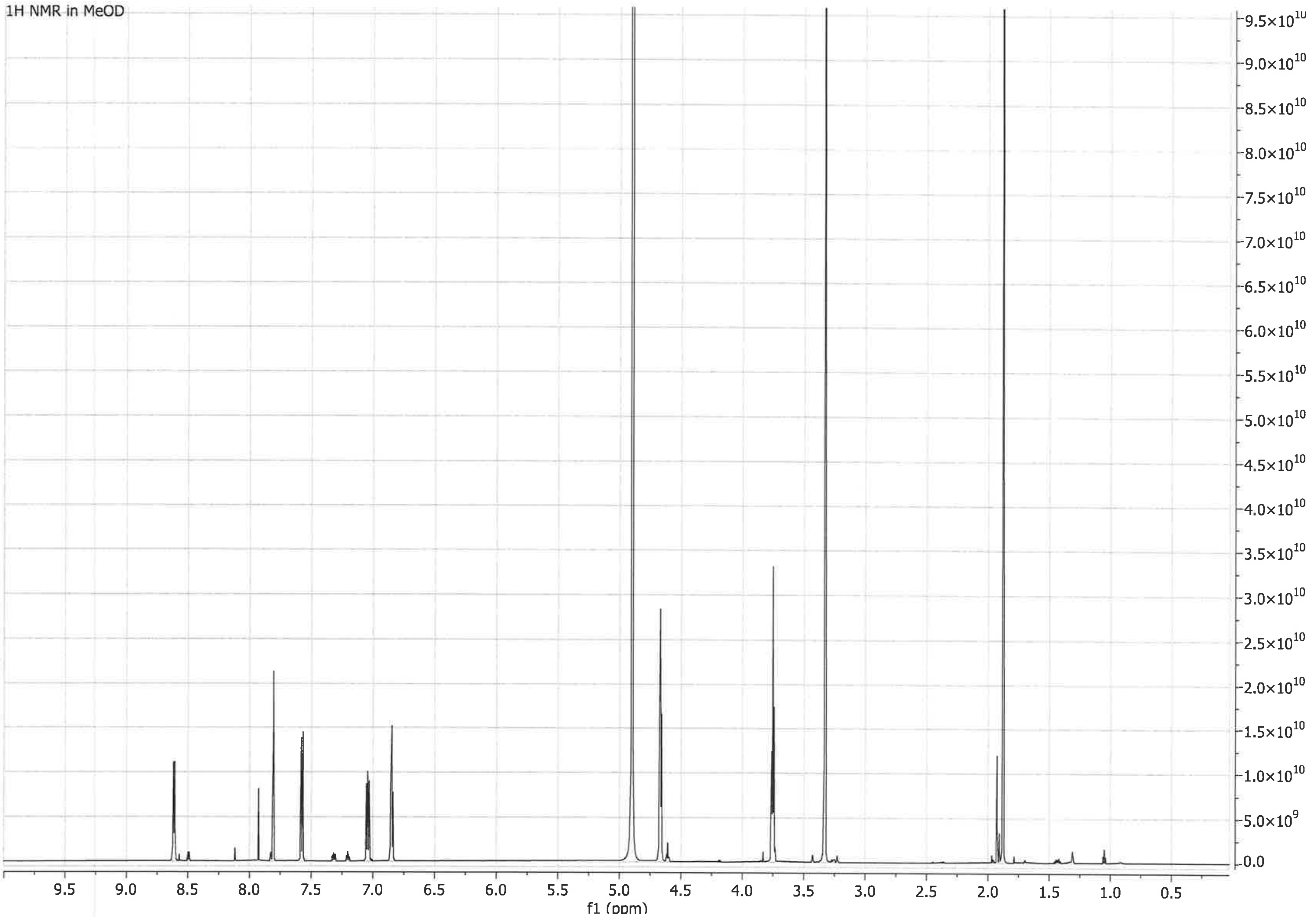
1H NMR in MeOD
9.5×10^10
9.0×10^10
8.5×10^10
8.0×10^10
7.5×10^10
7.0×10^10
6.5×10^10
6.0×10^10
5.5×10^10
5.0×10^10
4.5×10^10
4.0×10^10
3.5×10^10
3.0×10^10
2.5×10^10
2.0×10^10
1.5×10^10
1.0×10^10
5.0×10^9
0.0
9.5 9.0 8.5 8.0 7.5 7.0 6.5 6.0 5.5 5.0 4.5 4.0 3.5 3.0 2.5 2.0 1.5 1.0 0.5
f1 (ppm)

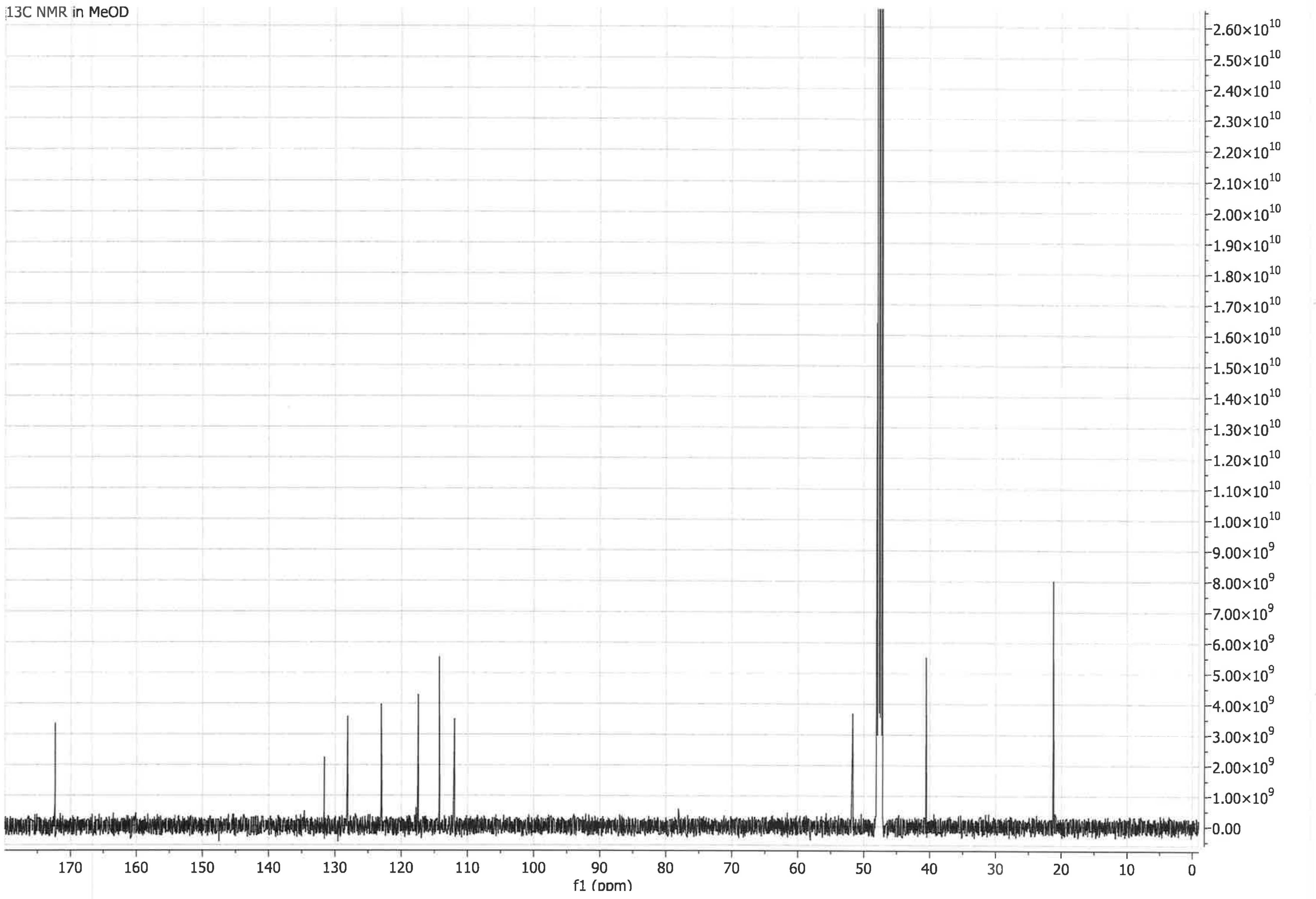
13C NMR in MeOD
2.60×10^10
2.50×10^10
2.40×10^10
2.30×10^10
2.20×10^10
2.10×10^10
2.00×10^10
1.90×10^10
1.80×10^10
1.70×10^10
1.60×10^10
1.50×10^10
1.40×10^10
1.30×10^10
1.20×10^10
1.10×10^10
1.00×10^10
9.00×10^9
8.00×10^9
7.00×10^9
6.00×10^9
5.00×10^9
4.00×10^9
3.00×10^9
2.00×10^9
1.00×10^9
0.00
170 160 150 140 130 120 110 100 90 80 70 60 50 40 30 20 10 0
f1 (ppm)

Mass spectrum of 2-pyridin- N-(2-ethylacetylamido)-2-yl-2-imidazole silver (I)Hexafluorophosphate (Ag5B(

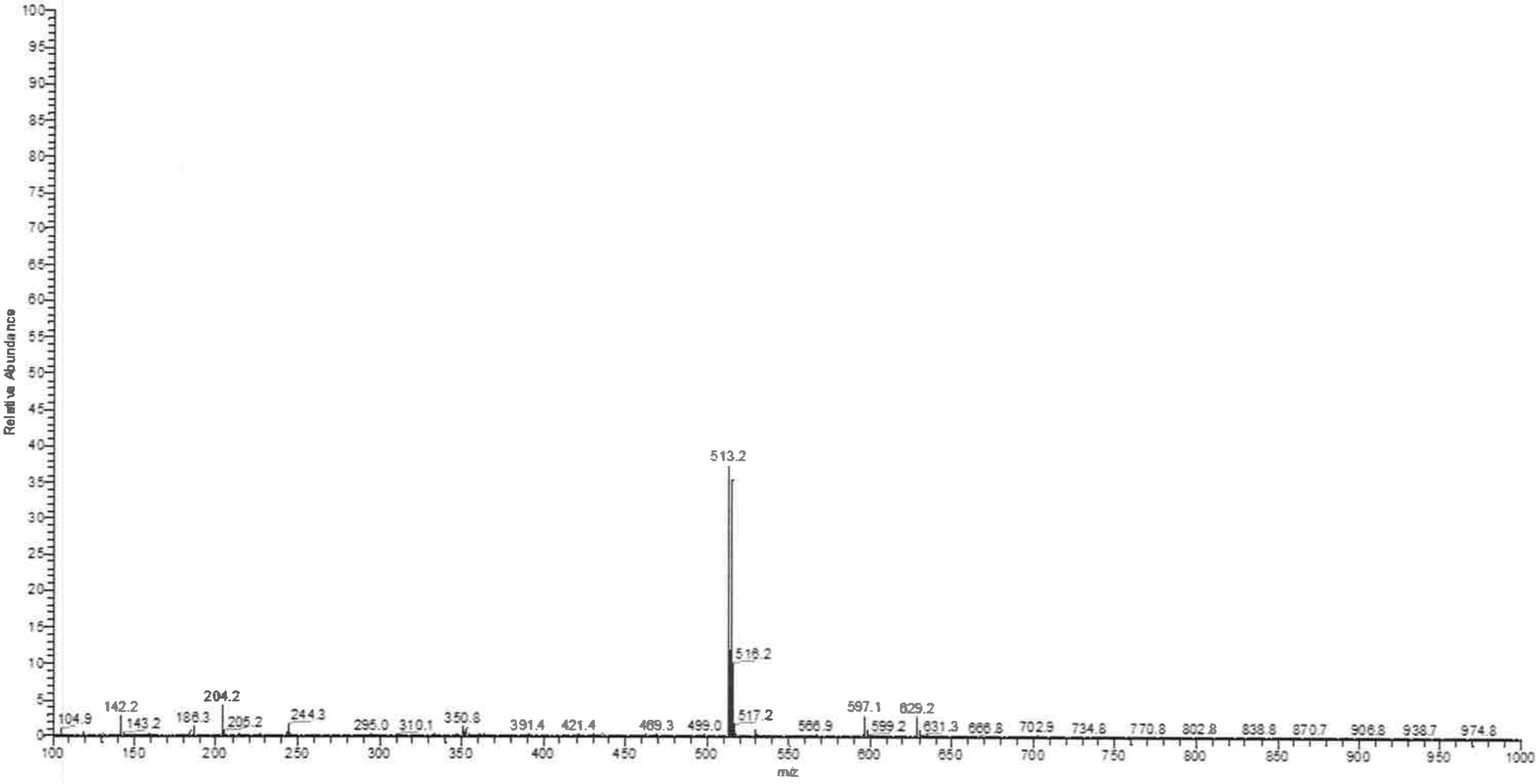

IR spectrum of 2-pyridin- N-(2-ethylacetylamido)-2-yl-2-imidazole silver (I)Hexafluorophosphate (Ag5BC)

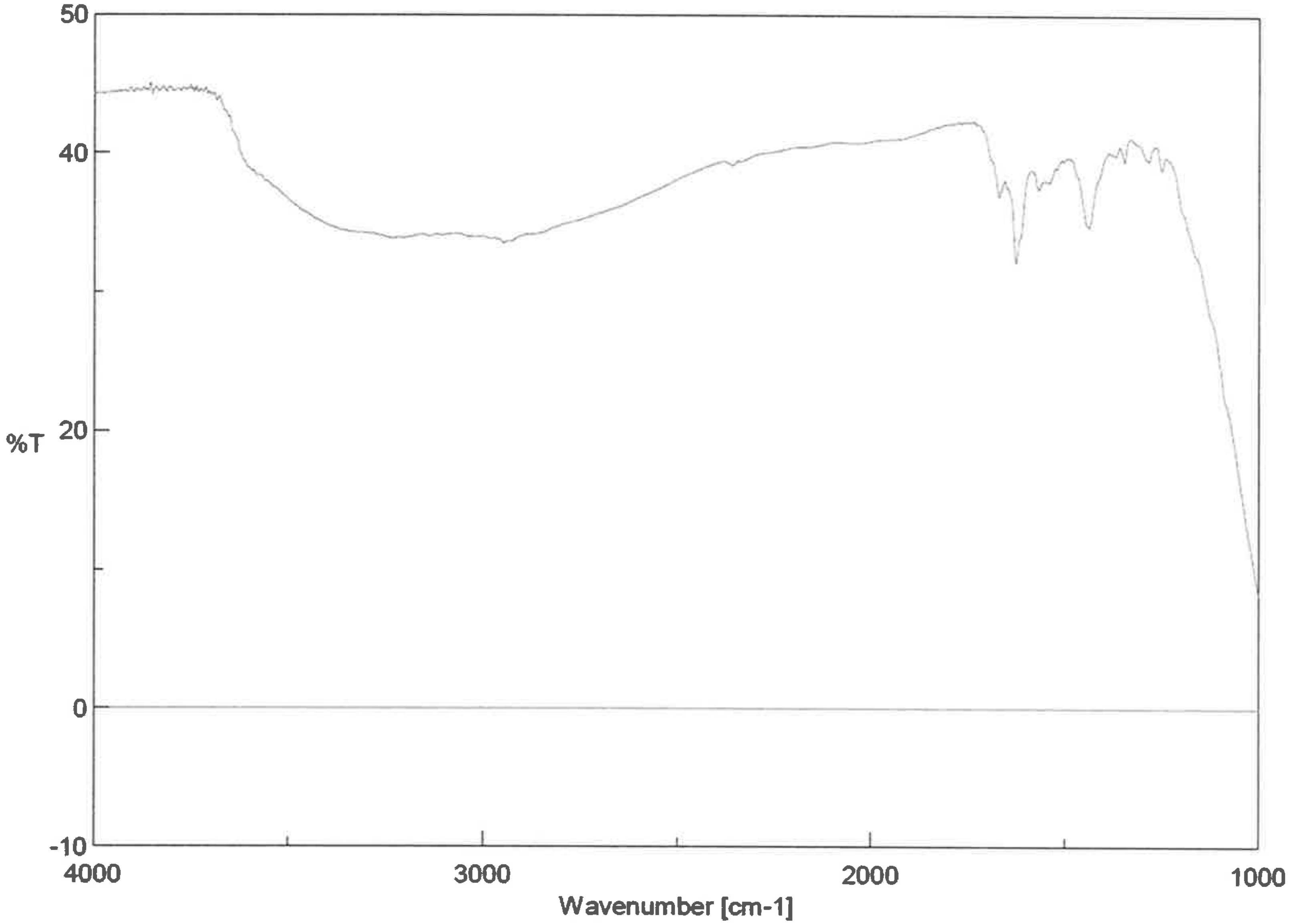

1H NMR in MeOD

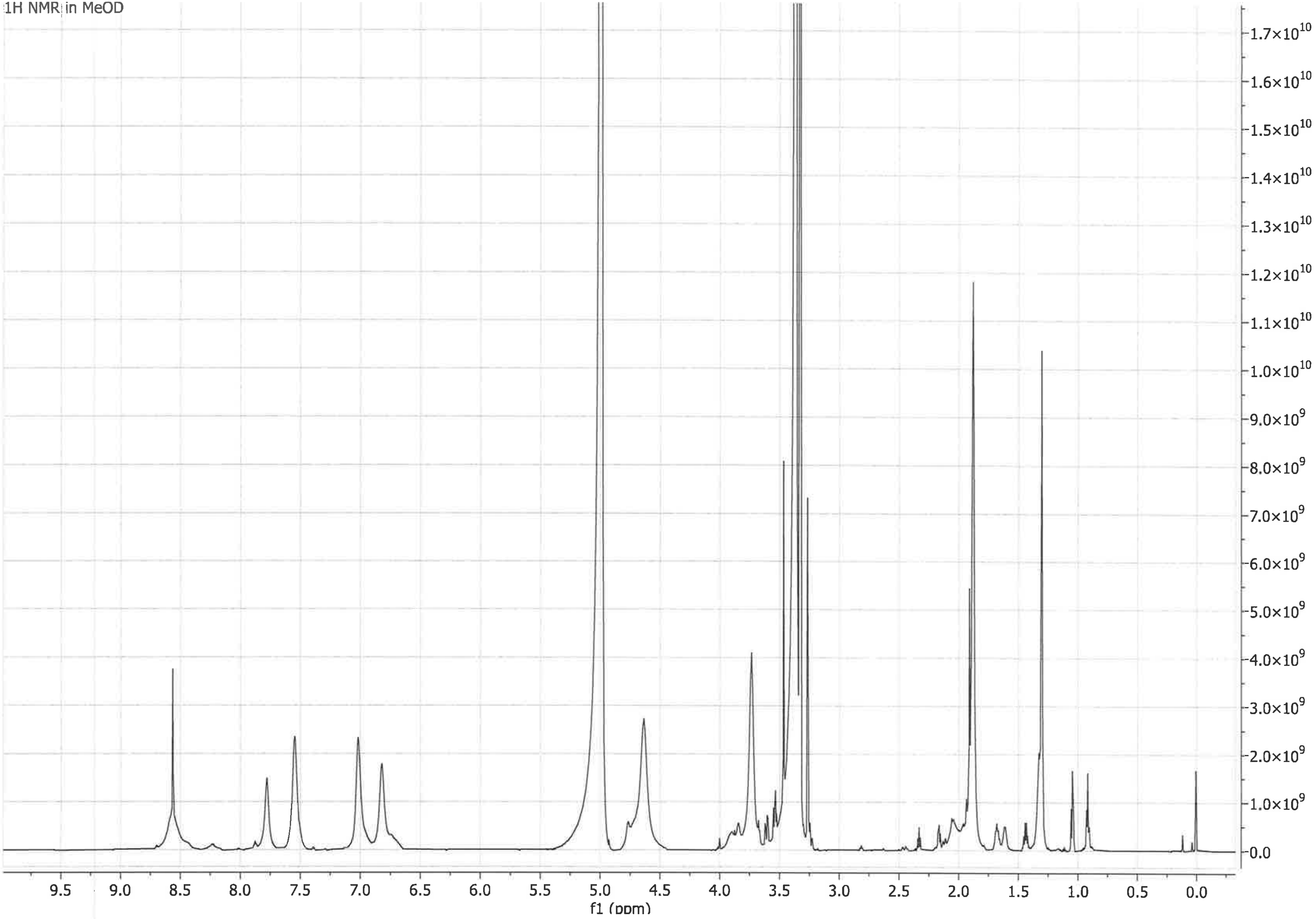

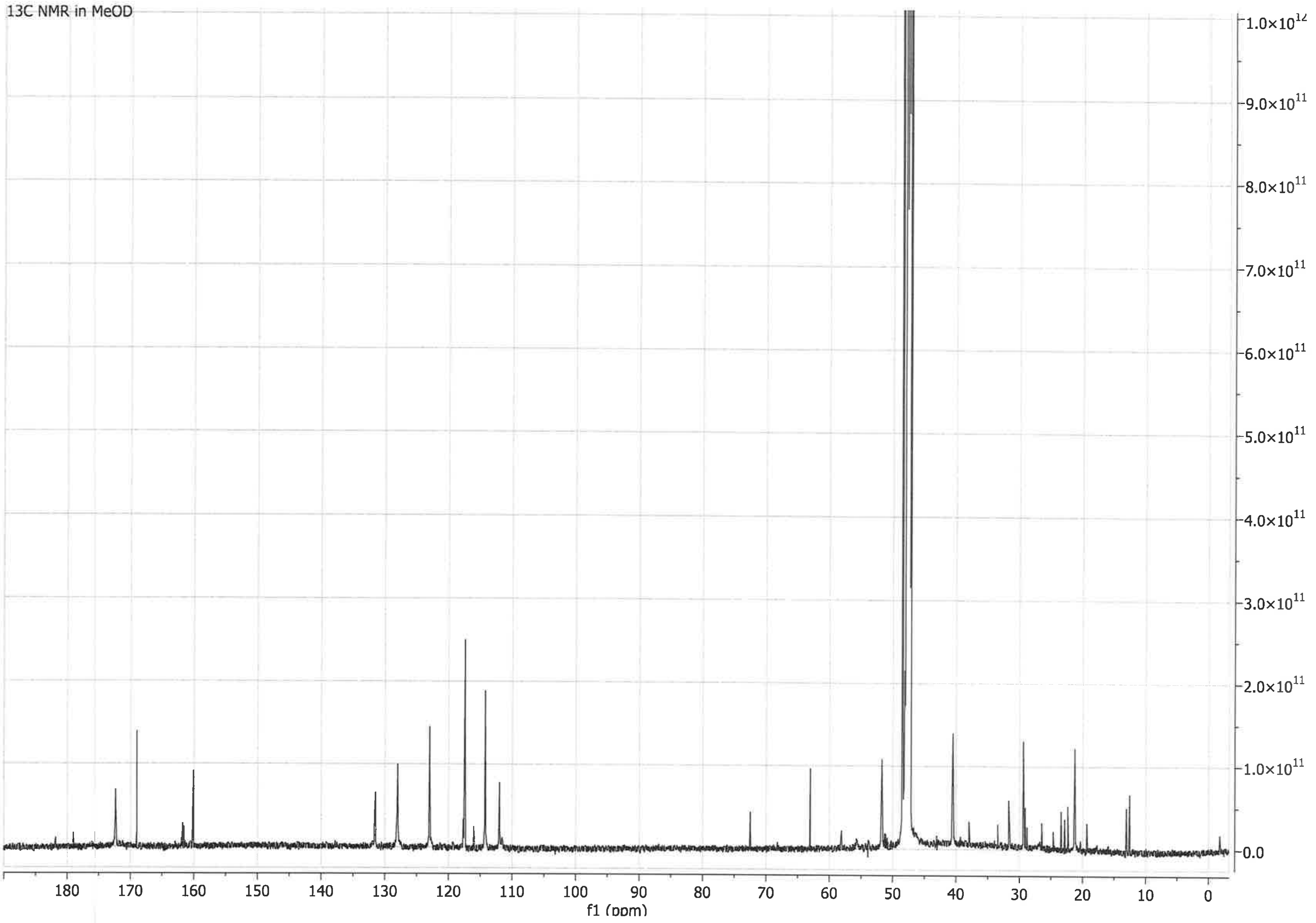
13C NMR in MeOD
1.0×10^12
9.0×10^11
8.0×10^11
7.0×10^11
6.0×10^11
5.0×10^11
4.0×10^11
3.0×10^11
2.0×10^11
1.0×10^11
0.0
180 170 160 150 140 130 120 110 100 90 80 70 60 50 40 30 20 10 0
f1 (ppm)

Mass Spectrum of Ag4MC

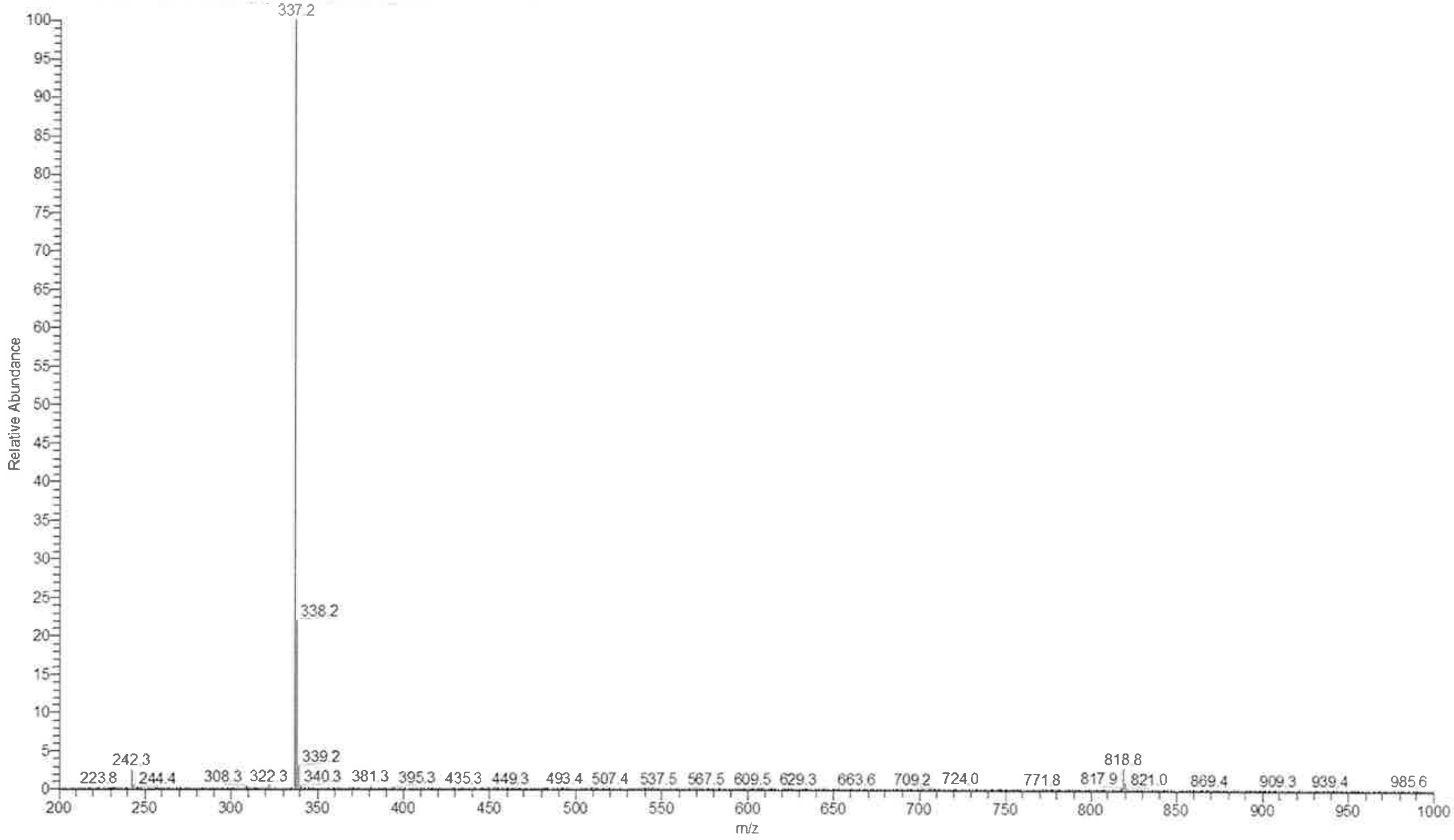

^{1}H NMR Ag4MC

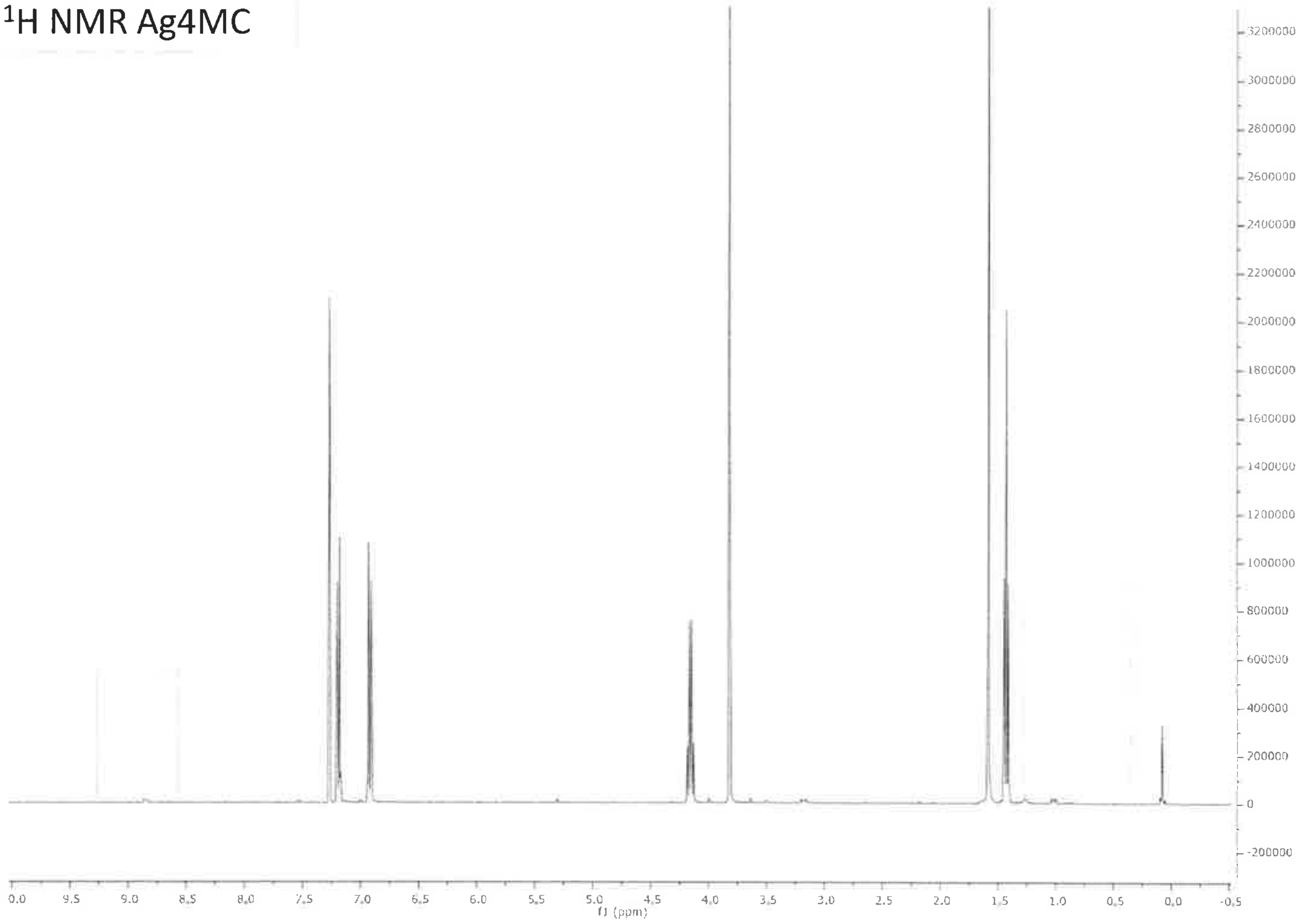

10.0
9.5
9.0
8.5
8.0
7.5
7.0
6.5
6.0
5.5
5.0
4.5
4.0
3.5
3.0
2.5
2.0
1.5
1.0
0.5
0.0
-0.5
f1 (ppm)
3200000
3000000
2800000
2600000
2400000
2200000
2000000
1800000
1600000
1400000
1200000
1000000
800000
600000
400000
200000
0
-200000

Mass Spectrum of Ag4BC

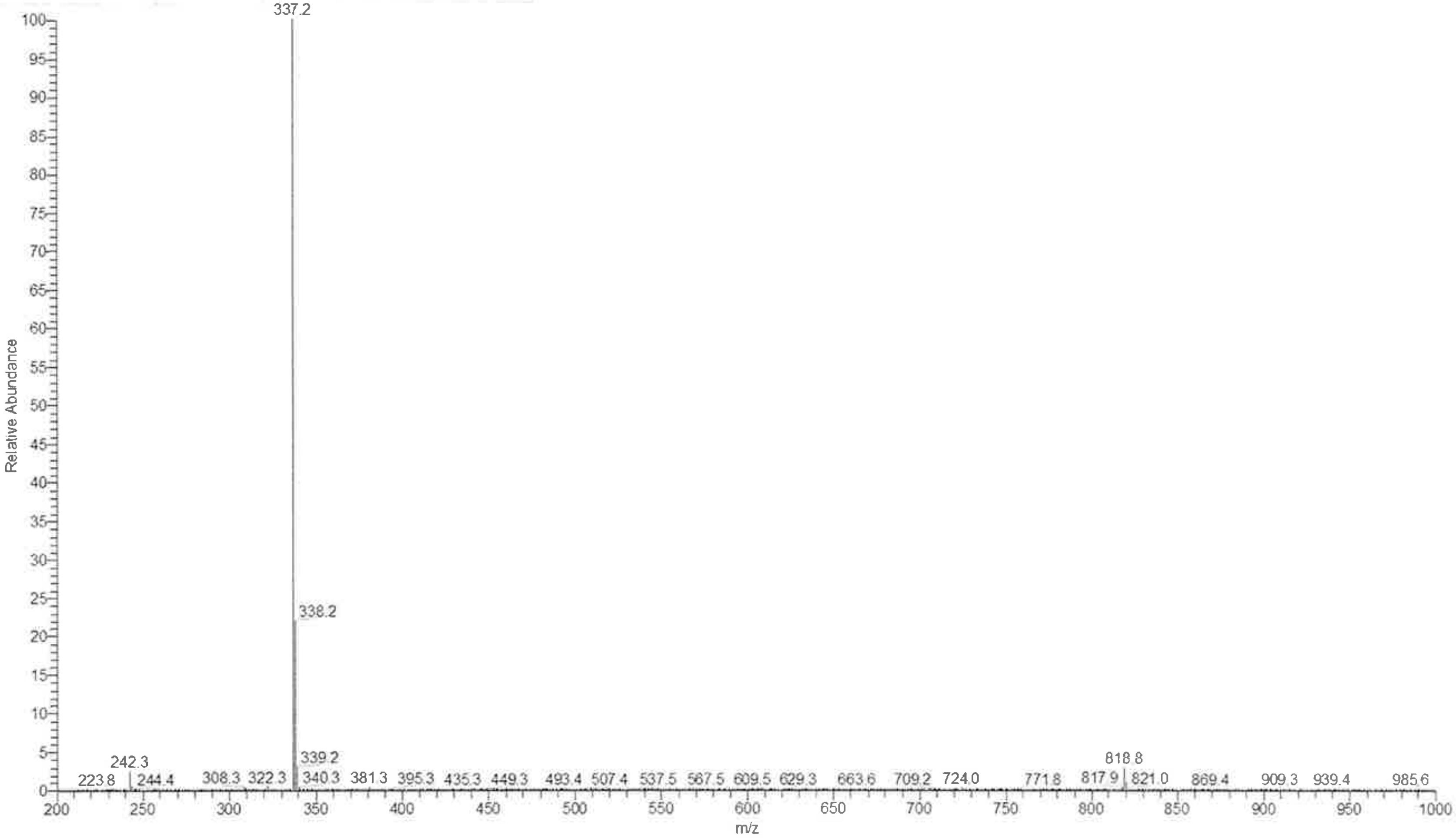

^{1}H NMR Ag4BC

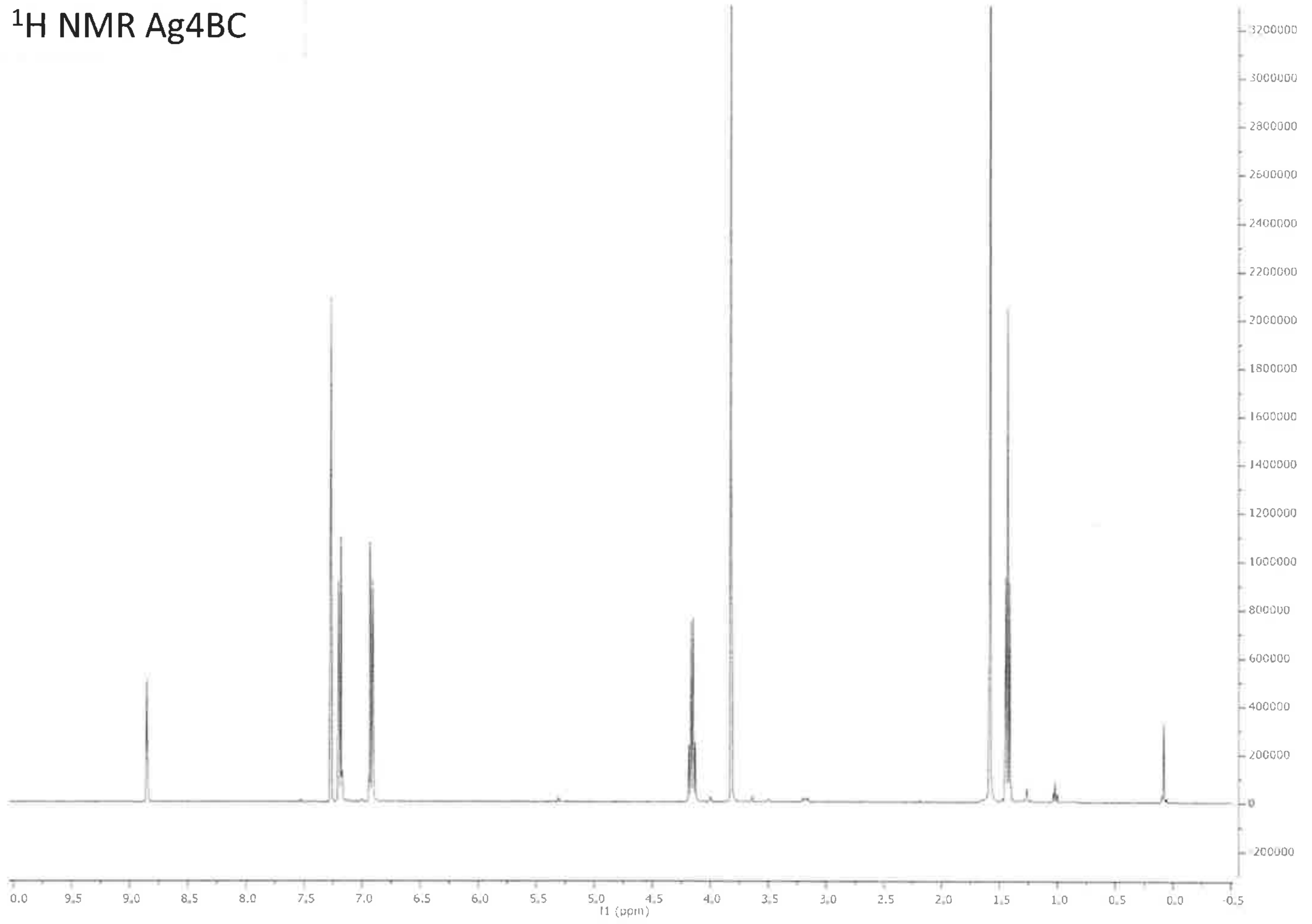

3200000
3000000
2800000
2600000
2400000
2200000
2000000
1800000
1600000
1400000
1200000
1000000
800000
600000
400000
200000
0
-200000
0.0 9.5 9.0 8.5 8.0 7.5 7.0 6.5 6.0 5.5 5.0 4.5 4.0 3.5 3.0 2.5 2.0 1.5 1.0 0.5 0.0 -0.5
f1 (ppm)
